# Enhancing the feeding journey of the preterm infant in the NICU: the STAmmi VICINO pathway as a model of integrated and individualized neurodevelopmental care

**DOI:** 10.3389/fpubh.2025.1597256

**Published:** 2025-09-12

**Authors:** Cecilia Naboni, Raissa Francesca Costantino, Stefania Longo, Pierpaolo Servi, Sonia Trussardi, Maria Grazia Tuoto, Stefano Ghirardello, Simona Orcesi

**Affiliations:** ^1^Department of Brain and Behavioral Sciences, University of Pavia, Pavia, Italy; ^2^Child Neurology and Psychiatry Unit IRCCS Mondino Foundation, Pavia, Italy; ^3^Neonatal and Intensive Care Unit, Fondazione IRCCS Policlinico San Matteo, Pavia, Italy

**Keywords:** preterm newborn, neurodevelopment, early intervention, NICU, healthcare providers, family centered care, neurobehavior, feeding

## Abstract

**Introduction:**

Preterm birth remains a significant public health concern, with infants born before 32 weeks of postmenstrual age at a higher risk for various comorbidities and neurodevelopmental impairments. Feeding challenges are particularly prevalent in preterm infants due to the immaturity of their physiological functions. The neonatal intensive care unit (NICU) environment can exacerbate these vulnerabilities through unregulated sensory stimulation and limited parental contact.

**Methods:**

A multiprofessional focus group was conducted to identify roles and responsibilities among NICU healthcare providers to develop and implement an individualized, integrated, interprofessional, and multidimensional care protocol. The aim is to promote neurobehavioral and early feeding skills in preterm newborns in the NICU. The protocol was grounded in the Newborn Individualized Developmental Care and Assessment Program (NIDCAP) and European Standards of Care for Newborn Health, with a focus on minimizing stress, enhancing parental empowerment, and utilizing a cue-based feeding approach.

**Results:**

The resulting “STAmmi VICINO” pathway consists of six flexible joint meetings at the infant’s crib, involving parents and healthcare professionals, to provide comprehensive care strategies for the transition from enteral nutrition to autonomous sucking. Preliminary integration in the NICU practice demonstrated the feasibility and positive acceptance of the protocol among practitioners.

**Discussion:**

Oral feeding skills in preterm infants should not be assessed separately from neurobehavioral development. An interdisciplinary, family centered approach is critical for successful early intervention programs that aim to positively impact neurodevelopment.

## Introduction

Preterm birth still represents, and increasingly so, a significant public health concern. Epidemiological studies have shown a persistent rise in premature births in both less developed nations with inadequate childbirth facilities and medium-to high-income countries. In these regions, factors such as advanced maternal age, multiple gestations, and fertility treatments are increasingly prevalent. Currently, about 1 in 10 neonates is born before 37 weeks of gestation (1). Babies born before 32 weeks gestational age, accounting for 15% (2), are at a higher risk of various comorbidities. Very preterm infants [VPT] face significant risks of complications, such as intraventricular hemorrhage, respiratory distress syndrome, infections, and neurodevelopmental and behavioral impairments, as the incidence of these complications generally decreases with increasing gestational age (3–5). Multiple organ systems may be affected by preterm birth, including those responsible for respiratory function, cardiovascular performance, immune competence, visual development, gastrointestinal function, endocrine regulation, and neurological maturation. The central nervous system is indeed particularly susceptible, as it is undergoing a critical phase of neuronal organisation and proliferation (6). Consequently, preterm infants exhibit increased vulnerability to structural alterations and brain injury. Recent studies have shown that preterm births have a much higher risk than term births of experiencing short- and long-term complications, proportional to the severity of prematurity and concomitant problems (7). In the long term, disorders can affect various developmental domains, such as language and communication (8, 9), fine and gross motor development (10), emotional-behavioral (11) and neuropsychological. Sequelae therefore may include cerebral palsy (12), severe developmental delays and complex neurosensory disorders (13, 14), as well as neurobehavioral and neuropsychiatric disorders (15). In contrast, in the short term, complications associated with the physiological immaturity of systems necessitating specialized and intensive care are frequently observed. The respiratory, cardiovascular, endocrine-metabolic, thermoregulatory, and gastrointestinal systems are most frequently subject to complications. These physiological systems, which are not yet fully matured at birth, are susceptible to substantial impairments, partly attributable to environmental exposure. The incomplete development of these vital functions at the time of delivery renders them particularly vulnerable to considerable disturbances, such as necrotizing enterocolitis (NEC), late-onset sepsis (LOS), and bronchopulmonary dysplasia (BPD). These conditions often occur simultaneously, complicating treatment and leading to poor outcomes. While life-saving, the artificial environment of the Neonatal Intensive Care Unit (NICU) plays a crucial role in the adaptation process of preterm newborns to extrauterine life. Necessary and frequent invasive medical procedures, unregulated sensory stimulation, and limited parental contact can potentially exacerbate existing vulnerabilities (16). Within this completely different environment, preterm newborns must adapt to gravity, the absence of physical confinement, and the transition from placental to pulmonary gas exchange. Furthermore, they must develop the capacity to process unfiltered sensory input that was previously attenuated by the maternal uterus, thermoregulate themselves, and reach autonomous feeding. Feeding challenges remain a persistent and increasingly prevalent issue in the preterm birth population, while simultaneously being frequently underestimated. Nevertheless preterm newborns, particularly those with low gestation age and low birth weight and/or with comorbidities, present a higher risk of feeding-related difficulties, mainly due to immaturity of physiological functions, including the ability to coordinate sucking, breathing and swallowing mechanisms (SSR) (17). An estimated 40% of preterm births have difficulty transitioning from gavage to oral feeding (18). Invasive medical procedures associated with preterm birth may also further impair the infant’s feeding behavior by failing to ensure adequate nutritional supplies, delaying the initiation and progression of full oral feedings (19–21), decreasing opportunities for positive oral experiences, and altering feeding-related experiences (22), particularly in VPT newborns (23). Research suggests that intense sensory experiences in the NICU may have devastating effects on the neurodevelopmental outcomes of premature infants. Stronger stressors include light, sound, and caregiving interventions (24, 25), such as medical and nursing practices that often include painful stimuli (26). Preterms show behavioral stress responses, such as jerking, twitching, arching, finger splay, leg extension, kicking, and arm waving, as well as stress physiological signals, such as changes in heart rate, respiratory rate, blood pressure, and oxygen saturation (26) in cases where sensory stimuli are excessively intense, frequent, prolonged, or complex (27). While research continues to investigate the specific types and quantities of stimuli that adversely affect the development of skills in preterm newborns (28), it is imperative to identify these patterns and develop early intervention protocols to mitigate their impact on their neurodevelopment.

## Aim

This project aims to define, develop, and implement an individualized, integrated, interprofessional, and multidimensional care protocol within the Neonatal Intensive Care Unit (NICU) setting. The protocol itself aims to promote neurobehavioral and early feeding skills in preterm newborns, with particular attention to very preterm newborns, who are most vulnerable to neurodevelopmental and feeding challenges.

## Methods

### Project setting

The project was created to develop a personalized, multidisciplinary intervention protocol for use in the Neonatal Intensive Care Unit of the IRCCS Fondazione Policlinico San Matteo in Pavia (Italy). This protocol aims to support premature infants and their families through a crucial and sensitive period, and its creation was driven by the department’s desire to effectively engage parents, enhance their understanding of their child’s medical status, and increase their involvement during the hospital stay. The goal was to share strategies and information to encourage parental care of infants. Additionally, the protocol addresses communication challenges arising from frequent staff rotations and promotes a holistic approach that extends beyond medical and nursing care to include neuroprotective care, recognizing the infant as a sentient and communicative being. Ultimately, this project seeks to contribute to interdisciplinary training aimed at enhancing implemented care practices. The operational framework for this protocol is NutriMenti, a multidisciplinary research project that aims to establish evidence-based operational protocols in the NICU. These protocols are designed to assist newborns, families, and medical and nursing teams in developing the oral nursing skills necessary for growth, development, and eventual discharge from the NICU.

### Theoretical framework

The theoretical framework of this quality improvement initiative is grounded in the NIDCAP program and derives its application from the European Standards of Care for Newborn Health published by the European Foundation for the Care of Newborn Infants (EFCNI). A review of these two documents was conducted to identify the key components of the protocol, assuring an evidence-based approach to the topic. The NIDCAP method is highlighted for its particular focus on infants’ individual traits and the active participation of caregivers. This approach begins with an evaluation and observation of the infant’s abilities and vulnerabilities, examining their level of stability and organization in accordance with Als’s synactive theory (29). Based on the identified neurobehavioral profile, a tailored care plan is developed for the infant and their family (30). This approach is founded on three fundamental principles: (1) infants are considered unique individuals who actively engage in their own care and receive nurturing support from their parents; (2) the primary role of parents as caregivers, protectors, and advocates for their babies is acknowledged, along with their involvement in care-related decision-making; and (3) healthcare professionals collaborate with infants, parents, and other family members to deliver comprehensive care services. The European Standards of Care for Newborn Health (31) were instrumental in identifying key areas of intervention for protocol implementation, ensuring early, personalized, and neurodevelopmentally oriented care. Particular emphasis was placed on the “care procedures” and “child- and family-centered developmental care” sections of the document. These sections outline strategies and interventions aimed at fostering infant development by minimizing perceived stress (such as postural care, sleep protection, hygiene procedures, and pain management), creating a positive environmental impact on the infant’s neurodevelopmental outcome (such as sensory stimulus regulation), and enhancing parental empowerment (including promoting parental involvement, case management, and discharge planning). Another key element of the theoretical framework is the “cue-based feeding” model. It represents a more sensitive and individualized approach than the less recent “volume-driven” model. It focuses on an infant’s ability to self-regulate feeding through behavioral and physiological cues. This method recognizes the infant as an active participant in the feeding process, allowing caregivers to adjust the rhythm and flow of milk according to the infant’s individual needs. Parents, in particular, play a key role in co-regulation, learning to recognize and respond appropriately to infants’ cues, thus promoting a positive and safe oral experience. The adoption of cue-based feeding involves several targeted strategies, including controlling milk flow to protect immature functions, avoiding excessive stimulation, maintaining appropriate posture, and stopping feeding at the first signs of fatigue. This methodology has been shown to reduce the time required to achieve independent oral feeding, shorten the hospital stay, and improve the dyadic relationship between the parent and infant (32). In light of this evidence, our research project draws on cue-based feeding as a role model, aiming to promote feeding practices that respect the neurobehavioral development of preterm infants and support their self-regulation. Effective implementation of this approach requires appropriate training of healthcare staff and careful communication among care team members to ensure safe and individualized care for each infant.

### Healthcare providers: who, what and why

**Figure 1:**
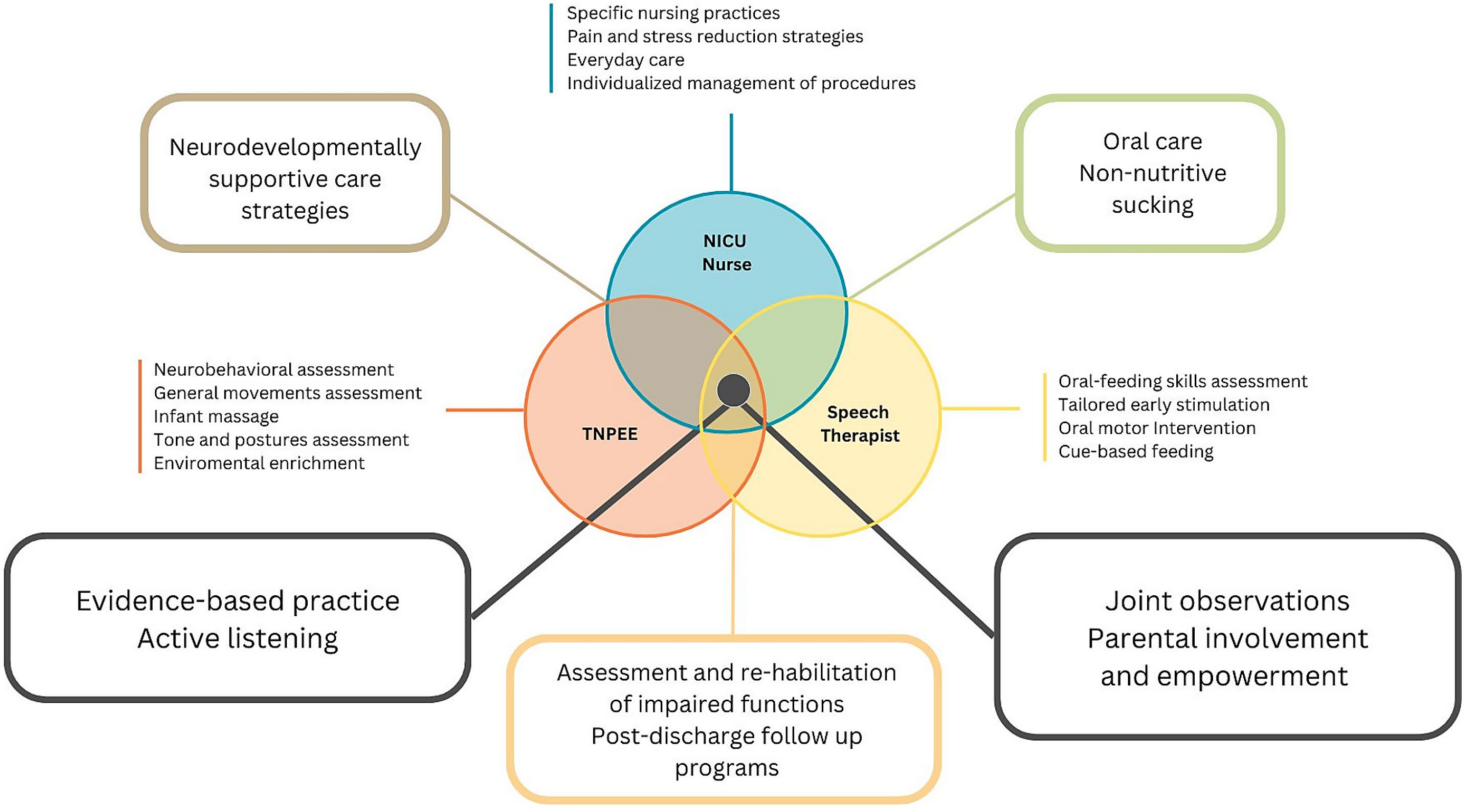
Schematic representation of the main responsibilities, roles, and activities of the three health professions involved in the project: NICU nurse, Speech Pathologist and TNPEE.

A multidisciplinary approach to preterm infant care is essential, given the complexity of preterm newborn development and care needs. Within our unit, a structured multiprofessional focus group was conducted including trained medial staff (neonatologists), NICU nurses and neonatal therapists. A purposive sampling strategy was used to involve professionals with direct experience in neonatal care. The group met across several moderated sessions using a semi-structured guide, aimed at mapping professional roles, identifying clinical priorities and integrating neurodevelopmental principles into a shared care pathway (Figure 1). Discussions were documented and synthesized to support the design of an operational and adaptable care protocol.

NICU nurses play a critical and multifaceted role in caring for sick newborn infants. They are responsible for providing complex and highly skilled nursing care, often serving as primary caregivers who offer constant attention to infants and their families (33). In addition to nursing practices, nurses are responsible for accompanying parents throughout the hospitalization period to learn and manage daily care activities, such as feeding, diaper changing, and bathing. Like other ward professionals, they are responsible for promoting newborn care practices aimed at reducing stress and pain and fostering a positive environment. A recent study by Brødsgaard et al. (34) highlighted the critical role of nurses in infant care and parental support for premature babies. Nurses are tasked with establishing an environment that facilitates parents’ learning and skill development in caring for their preterm infants. This involves motivating and enabling parental participation in infant care and guiding them from passive observation to active caregiving. Essential aspects of the nurse’s role include effective communication and information dissemination, with regular meetings and in-depth discussions promoting parental recognition and involvement. Nurses cultivate trust in parents through transparency, sincerity, and acknowledgment of parental experiences. They offer personalized support tailored to each family’s requirements and emotional state while guiding parents in enhancing their parenting abilities and self-assurance over time. As the infant’s health improves and parents become more proficient, nurses engage in role negotiations, gradually empowering parents to assume greater responsibility for their infant’s care. This evolving process necessitates continuous adjustment of the nurses’ approach, balancing their expertise with the aim of fostering parental autonomy and confidence in caring for their premature infants.

The Language and speech pathologist is specialized in the prevention and early treatment of disorders of oral, eating, and communication function (35). Within a cue-based feeding approach, the speech therapist plays a key role in the transition to safe and effective oral feeding in preterm newborns. The intervention is structured through a functional assessment of the infant and the implementation of oral stimulation strategies, with the goal of facilitating the transition from non-nutritive to nutritive sucking. Within the NICU environment, the therapist evaluates the infant’s oral anatomical structures and functional development using both visual and tactile feedback. Treatment is not limited to the immediate management of difficulties but takes on an important preventive function, reducing the risk of swallowing, speech, and communication disorders. Parent education is another pillar of speech therapy intervention, as it enables parents to acquire fundamental skills to optimize their relationship with their infant and foster secure attachment. In addition, speech therapists participate in post-discharge follow-up, monitoring the child’s development and contributing to the prevention of neurodevelopmental sequelae through personalized interventions.

The Neuro and Psychomotor Therapist of Developmental Age (TNPEE) is a specialized profession focused on creating and implementing preventive programs, as well as assessing and rehabilitating individuals in the developmental age (36). This is achieved through a holistic approach that evaluates both neurological and psychomotor aspects. Drawing on contemporary neuroscience, the TNPEE designs interventions around children’s spontaneous behaviors, taking into account their developmental stages, surroundings, and the roles of caregivers. This adaptable and ecological method incorporates the latest neuroscientific findings on brain plasticity and neurodevelopmental disorders. TNPEE practitioners work in close cooperation with other pediatric specialists in multidisciplinary teams, offering neurodevelopmental enhancement techniques and therapeutic education to families. In neonatal intensive and sub-intensive care units, these professionals play a crucial role in multidisciplinary teams that care for premature infants and newborns with complex medical conditions. They employ early assessments and intervention strategies, enhance neurodevelopmental outcomes, provide an individualized sensory experience, and support parents in understanding their newborn’s cues and bonding with them during hospital stays (37).

## Results

### STAmmi VICINO pathway

**Figure 2:**
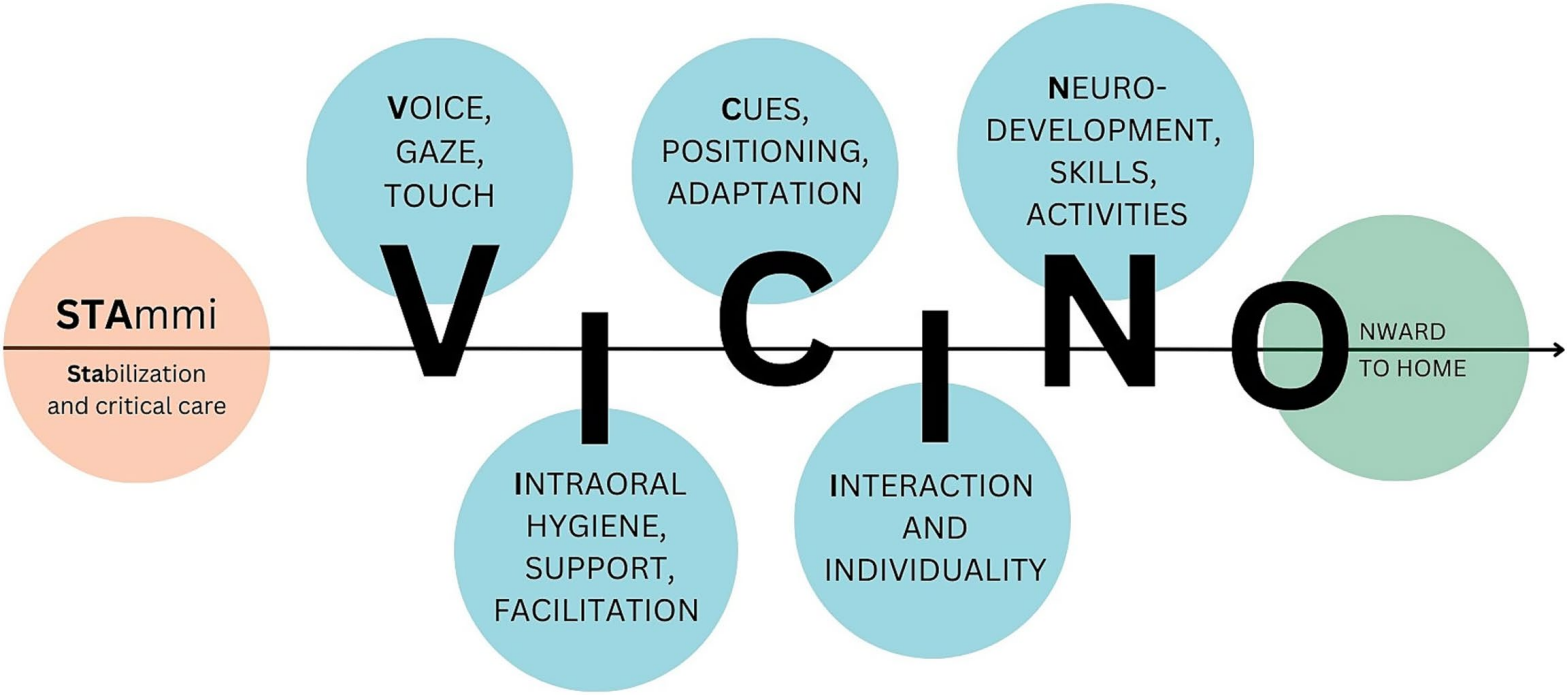
“STAmmi VICINO” pathway.

Multidimensional and multiprofessional perspectives and guidelines on neurobehavioral development, oral feeding skills, and family centered care in the NICU setting were considered during the protocol’s development. As aforementioned, the early intervention protocol is designed to be multidisciplinary, flexible, and customizable, and is adaptable to the individual needs and characteristics of each infant and family. The pathway aims to accompany and support patients and families during hospitalization through targeted team intervention, fostering the creation of a stable and positive triadic bond. Based on these considerations, we established the “STAmmi VICINO” pathway (translated as “Stay Close to Me”; Figure 2), wherein each letter represents a distinct phase of the early intervention protocol.

This six-step pathway supports the entire family from the first weeks through discharge, providing comprehensive, integrated, and multidisciplinary care strategies for the transition from enteral nutrition to autonomous and effective sucking for nutritional purposes. It includes six (flexible) joint meetings at the infant’s crib, in the presence of the parents, referring nurse, and neonatal therapist, to facilitate the transferability and ongoing application of the intervention. The domains encompassed within this pathway include the facilitation of early parent-infant interactions through eyes, voice, and touch; the provision of support and enhancement of early oral skills; the assessment of newborn behavior; the implementation of postural care strategies; the promotion of continuous and early interaction and fostering of individuality; the observation of emerging neurodevelopmental skills; and the facilitation of effective oral feeding skills. Based on what is observed at each time point, individualized strategies are shared with parents and professionals in both oral and written forms.

#### Stabilization and intensive care

During the initial phase, when the clinical conditions are most critical, the primary referral figures for the family are the neonatal physician and the NICU referral nurse. At this stage of the protocol, no direct interventions are implemented on the infant; however, standard care is provided to promote harmonious development, prevent complications associated with preterm birth, and minimize the impact of the NICU environment on the involved parties (infant and family).

#### Voice, gaze, touch

During the first joint observation, conducted after the infant’s clinical condition has stabilized and preferably between 28 and 30 weeks of postmenstrual age, TNPEE and nurses accompany the parents in observing the infant’s spontaneous behavior. The first joint observation is performed using the Preterm Infant’s Assessment for Neurobehavioral Observation (PIANO), developed by Naboni C. and colleagues (unpublished). The goal is to obtain an initial assessment of the neurobehavioral stability of the preterm infant in relation to all domains analyzed by the scale: neurovegetative stability, postural skills, spontaneous motility, response to interaction, attention status, and early visual skills. During the joint observation, the TNPEE provides early positive sensory experiences; moreover, early interactions between the parent and infant are encouraged and supported by the healthcare providers: parents are accompanied in recognizing behavioral states, signs of readiness or unwillingness to interact, making contact with their baby first with their gaze, then with their voice, and finally with their touch. At the end of the first joint observation and in the following days, health professionals involved, especially nurses, propose positive sensory experiences, such as Kangaroo Care, based on the characteristics, habits and needs of the family (infant and parents).

#### Intraoral hygiene, support, facilitation

The second joint observation is carried out subsequently, with a timeline depending on the clinical development of the infant. The speech therapist, in co-partnership with the TNPEE and the ward nurse, observes the presence of orally directed behaviors and signs of readiness/unreadiness for feeding, makes initial guided observations of early oral positive experiences, and, if necessary, proposes individualized oral stimulations, also involving the parents in their daily implementation. The therapist assesses the infant’s oral structures, tongue mobility, and functional development using visual and tactile feedback. During sucking and bottle feeding, the coordination of the cheeks, jaw, and lips and the rhythm of sucking, swallowing, and breathing are observed, monitoring for issues such as choking or oxygen desaturation. Among the tools used for the assessment of oral feeding skills, Premature Infant Motor Intervention (PIOMI) (38) is proposed: a 3-min long eight-step protocol created specifically for the preterm infant population. Through this assessment, the state of activation of the peri-oral area muscles and the infant’s ability to activate or not activate effective non-nutritive sucking can be checked. During the assessment, stimulation is applied within a cue-based, neuroprotective framework and sessions are interrupted or adjusted at the first sign of stress. Based on what has been concluded, caregivers and nurses are accompanied in understanding and experimenting with the most appropriate oral stimulations for that specific infant, promoting not only their active participation but also the transferability and continuity of the intervention. Alongside the speech therapist, the TNPEE assesses the newborn’s ability to reach and maintain a sufficient alert time, which is a key element for feeding readiness. The objective of the second joint meeting is to conduct a specific assessment and provide the basis for the initiation of effective oral feeding.

#### Cues, positioning, adaptation

The third joint meeting aims to assess the developmental progress of premature infants and evaluate the behavioral responses observed during recent procedures or positional changes. Based on the infant’s developmental stage and acquired abilities at this meeting, guidance and techniques will be provided by the TNPEE in collaboration with other healthcare professionals. These may include interpreting signs of comfort or distress, maintaining proper posture, preventing skull deformities, and methods for soothing the infant during medical or nursing interventions. The focus of the meeting is to share practical strategies for coping with daily activities: *how to “stay close” during a medical-nursing procedure? How can we move through space while continuing to foster postural alignment? How do interactions between parents and children evolve? How can visceral and motor stress signals be interpreted? How can development be facilitated during interactions with caregivers?*

#### Interaction and individuality

In the fourth joint observation, the speech therapist takes over again, alongside the TNPEE and the referral nurse, who accompanies the infant and parents during breastfeeding or bottle feeding. At this stage, positions are identified together to facilitate mealtimes that are comfortable for both the infant and the parent; the type of bottle and flow so that they are appropriate to the infant’s skills and size; and caregivers are accompanied in reading the signals manifested by the infant during the meal and recognizing when breaks are needed. Moreover, feeding intolerance signals are assessed by NICU nurses. The goal of the meeting is to coordinate the professionals who revolve around the infant’s mealtime (nurse, neonatologist, TNPEE, speech therapist) so that they are aware of the infant’s parental attitudes, preferences, feeding, and postural needs. The key word of the meeting is *Individuality*: the professionals involved aim to adapt the recommendations and guidelines to daily clinical reality, without distorting them; parents are accompanied to recognize more effectively the infant’s responses, especially related to mealtimes, and to respond according to their own instincts, respecting the safety and autonomy of each of those involved.

#### Neurodevelopment, skills, activities

During the fifth joint observation, changes associated with the infant’s behavioral maturation are observed and emphasised: from the assessment of spontaneous motility to the postures adopted, progressing through curiosity and responsiveness toward the environment. The TNPEE supports parents in recognizing the various behavioral states and their alternation throughout the day, identifying the most favorable moments during which to initiate the first interactive exchanges, and experimenting with initial daily care activities such as nappy changing or bathing. These activities are proposed to facilitate positive sensory characteristics and minimize negative experiences, as they are potential sources of stress.

#### Onward to home

The sixth and final joint observation is conducted as discharge from the ward approaches when the infant’s clinical stability has been established. TNPEE, speech therapists, and nurses guide the family toward the transition home: information is provided to parents regarding common developmental supports available, and recommendations are made for appropriate bottles and pacifiers for their infant’s use at home. Additionally, any queries and concerns of caregivers are addressed, and the post-discharge developmental follow-up protocol is explained. During this session, parents are afforded the opportunity to practice recent caregiving techniques that they had not previously performed independently. Under staff supervision, they administer prescribed therapies, learn the proper use of infant carriers, and/or explore novel strategies for interactive play. Concurrently, a second neurobehavioral observation is conducted using the PIANO assessment tool, enabling the sharing of the child’s developmental progress and acquired skills with the family. The shared observation also serves to identify areas of persistent vulnerability. The TNPEE subsequently provides practical strategies for maintaining close engagement during the initial weeks at home, pending the first post-discharge evaluations.

### Preliminary integration in the NICU operational framework

Following the initial establishment of the intervention protocol, a preliminary integration of the model was undertaken within our neonatal intensive care unit protocols. The swift transition from theoretical framework to practical application was facilitated by the fact that all healthcare personnel involved in the protocol were already employed in various capacities, with some variability in terms of timesheets, within the standard care framework of the unit. Similar to the personnel, the tools incorporated in the protocol, namely the PIANO neurobehavioral scale and the oral-motor intervention protocol (PIOMI) (38), are integral to the NICU’s standard care. However, prior to the establishment of the STAmmi VICINO pathway within the NutriMenti project, these tools were not utilized in a coordinated or systematic manner. From July 2024 this model was used at our facility to sistematically implement clinical practice and to establish a multidisciplinary accompaniment in the neurobehavioral and oral feeding skills maturation pathway of preterm newborns. The project involved a TNPEE, a speech therapist, two nurse practitioners, medical and nursing staff on duty at crib meetings, and two neonatal therapists-in-training. This initial application of the pathway occurred during the final stages of its refinement and aimed to verify its applicability within standard care procedures.

During the testing period, the practitioners involved had the opportunity to confront each other both before and during each household’s journey, recognizing the need to share reflections, suggestions, and practical guidance not only with parents but also within the multidisciplinary team. Therefore, several thematic booklets were created and shared in two versions: (1) one for the family, containing simple but precise language, enriched with practical guidance, and (2) one for the practitioners involved, dedicated to sharing evidence-based strategies of neurodevelopmental care and promotion of oral feeding skills, including strategies deemed effective for that infant at that time. The material aimed to ensure continuity and coherence of care practices, facilitating the dissemination of the protocol’s content across the multidisciplinary team and promoting shared reference tools within clinical routines. Although no formal feedback was collected, parents spontaneously shared positive impressions during routine bedside meetings. Many expressed how helpful it was to recognize their infant’s subtle behavioral cues, which enabled them to better understand and respond to their baby’s needs, not only during feeding time, but also in daily interactions.

## Discussion

This project advocates for the definition and initial integration into clinical practice of a novel model of early, multidisciplinary, family centered intervention protocol aimed at supporting neurobehavioral and oral feeding development in preterm infants during their hospitalization in the NICU. Despite advances in intensive care medicine and care practices, preterm infants continue to be an extremely fragile population prone to numerous complications. Furthermore, the neonatal intensive care unit (NICU) environment does not provide sensory stimulation that aligns with developmental needs and exposes infants to daily sensory challenges. Feeding difficulties may become apparent during NICU admission, such as the transition from parenteral to tube feeding and progression to independent full oral feeding due to immature SSR coordination (39).

### The feeding pathway in the NICU: a greater challenge for preterm newborns or for healthcare providers?

Challenges in attaining autonomous oral feeding in preterm infants, coupled with the risk of inadequate nutritional intake, remain a subject of significant interest for NICU teams and the broader scientific community. These factors are recognized as contributors to the development of postnatal growth retardation, a condition known to substantially impact the duration of hospitalization, subsequent inpatient-related expenses, parental distress, and long-term neuropsychological outcomes (38, 40–43). Nonetheless, there remains a lack of consensus among clinicians and researchers regarding the assessment and treatment of feeding difficulties. Some studies (44, 45) have highlighted the lack of specific policies and guidelines for initiating suck feedings in preterm newborns, with providers primarily using behavioral cues, gestational age, and weight to assess readiness. Despite the passage of three decades, there remains a notable absence of validated, structured oral feeding guidelines for optimal management and assessment of feeding readiness and skills, resulting in considerable variation in practices among healthcare institutions (43, 46). This is also influenced by the qualitative/descriptive over quantitative/objective approaches for assessing preterm oral feeding performance (43). Nevertheless, recent research conveys that not only coordinated sucking, swallowing, and breathing are the key factors that can influence the preterm infant’s ability to feed efficiently, but also physiological stability, tone control, behavioral state organization, and neurobehavioral maturation (47). Although many tools and protocols exist for evaluating and promoting the feeding abilities of preterm infants (22, 42, 43, 47), their incorporation into neurobehavioral assessment instruments remains inadequate (48). One potential explanation for this issue is the insufficient systematic presence of specialized professionals, such as neonatal therapists, in Neonatal Intensive Care Units (NICUs) in Italy and globally. This is compounded by the limited knowledge and application of the latest evidence-based practices by nursing staff to support feeding. As noted by Thurin et al. (49), NICU nurses occupy a unique position for observing newborns and their characteristics because of the extensive hours they spend working next to the crib with babies and their parents. However, they still struggle to achieve sufficient knowledge of inherent cue-based feeding, oral-motor development interventions, feeding positions, and flow regulation during feeding (50). Some authors have explored the possibility of implementing the knowledge of healthcare providers, especially nurses, on the issue to achieve a positive effect on infant feeding skills, and the work of Beissel and colleagues (39) demonstrated the positive effect of an \"infant cue-based feeding bundle and nurse education program\” on the early achievement of independent oral feeding in preterms, confirming the feasibility and acceptability of the program among providers.

### The need for an interdisciplinary approach to early intervention

To the best of our knowledge, this study is the first to propose an intervention model designed to optimize the feeding pathway for preterm infants through a multidisciplinary and integrated approach utilizing early intervention and family centered care models within the NICU. In addition, our intervention model aims to implement the knowledge of health professionals working in the NICU through mutual information and sharing specialized, evidence-based practices. It also aims to foster parental empowerment through the early and ongoing involvement of family members. As highlighted in a recent review by Tang et al., the success of an early neuroprotective intervention protocol in the NICU is contingent upon close, ongoing collaboration between healthcare professionals and families, as well as specific training for staff involved in daily NICU care. The implementation of neurodevelopmental care practices, derived from Als’ Synactive Theory (29) and foundational intervention models as NIDCAP and SENSE (51), necessitates the integration of various professionals and the early and continuous involvement of the family in decision-making and operational processes. Within this framework, the way we approach a newborn and the way sensory input is offered to the infant becomes a crucial aspect of care, not only in terms of what is provided, but how and when it is delivered, based on the newborn’s cues and the dyadic interaction with the caregivers. In line with this model, our protocol adopts a comparable definition of appropriate sensory stimulation, understood as developmentally supportive, contingent and behaviorally guided multisensory input. This approach respects the infant’s readiness and self-regulation and aligns with the principles of individualized, neuroprotective care. In our clinical practice, this means adapting both the macro and micro-environment to support the newborn’s stability and engagement. During bedside interactions, professionals and parents jointly observe the newborn’s cues, and interventions are shaped accordingly, for example helping a caregiver adjust the tone of voice or type of touch based on the baby’s reactions. We emphasize a “one stimulus at a time” strategy to avoid overstimulation and promote a positive sensory experience. These principles are aligned with both the SENSE model and the foundations of cue-based feeding, reinforcing a shared clinical philosophy of individualized, responsive care. Regarding oral feeding assessment and intervention, a consensus among researchers (22) highlights the importance of initiating these practices at an early stage using valuable and standardized tools. Within our project, we decided to use Non-Nutritive Sucking (mainly performed by nurses) alongside the PIOMI (performed by the speech therapist) because of their ease of implementation in clinical practice, their applicability as early as 29 weeks PMA, and because they were found to be the most effective in improving feeding quality, achieving full oral feeding faster, and consequently reducing the duration of hospitalization (22). In our setting the PIOMI protocol is used as a flexible tool, integrated within a broader developmental framework that prioritizes the infant’s behavioral signals. We consider feeding as a social process within our protocol: eating for a newborn corresponds not only to the mere mechanism of swallowing the bolus and coordinating the mechanism of sucking, swallowing, and breathing, but is a complex process involving physiological and behavioral stability, as well as the relationship with the adult. Being fed is not a passive act but requires active participation on the part of the infant, which is why it is essential that oral-motor and functional skills are not the only ones considered. For these reasons, two key elements of our intervention protocol are the systematic, shared, and continuous observation of the neurobehavioral cues of the preterm infant and the active involvement of the parents, who are considered the best experts on their child within our clinical practice. Although structured neurobehavioral assessments are currently scheduled at two time points within our protocol, the constant presence of the TNPEE throughout the observations allowed for a joint analysis of the infant’s behavioral cues at all encounter occasions, allowing the proposed stimulations to be tailor-made and the activities proposed in the pathway to be adapted to individual needs. The literature (52) supports the effectiveness of interventions to support parenting skills in terms of reading the infant’s signals and responsiveness. We believe that our study can be the first step in establishing integrated protocols not only in transdisciplinary terms but also in reinforcing collaboration between health professionals and families. Based on our observations, the STAmmi VICINO protocol holds promise as a framework for guiding direct interventions for newborns and their families in the NICU. The flexibility inherent in the protocol, which does not mandate that meetings occur at a specific postmenstrual age, allows for the adaptation of the pathway to align with the child’s clinical progression and the family’s needs. In line with this, although the protocol was primarily conceived with very preterm newborns in mind, its adaptable design makes it suitable for broader application across the preterm spectrum, including late preterm newborns, depending on individual needs and developmental profiles. Its initial implementation in our unit has demonstrated its effectiveness as a supportive tool, enabling the customization of newborn care according to individual characteristics and clinical conditions by modifying both macro and microenvironments. Additionally, it aids parents in understanding and recognizing their baby’s behavioral cues, empowering them to become confident and informed caregivers, which fosters the development of a stable and positive emotional bond. Furthermore, it facilitated better collaboration among various professionals in the NICU and enhanced their participation in neurodevelopmental care practices. Our findings reinforce the idea that innovation in neonatal care does not necessarily require the introduction of new tools, but rather a systematic integration of existing practices within a shared, developmentally-supportive grounded framework.

## Limitations

Despite the potential of this protocol, several limitations remain. In particular, the part-time and unstructured availability of the healthcare professionals involved limited the extent to which the protocol could be applied more broadly during the preliminary integration. As this initiative was developed and carried out within standard care pathways, its scalability remains to be further explored. While it is not imperative for these specific professionals to be present within the NICU to establish a protocol with these objectives, the absence of trained personnel in behavioral assessment, in the implementation of neurodevelopmental care practices, and in the support of oral-feeding skills protocols can pose significant challenges to the protocol’s implementation in other NICUs. Furthermore, the substantial number of nurses and physicians in the NICU, coupled with continuous staff turnover, are potential impediments to maintaining clear and effective transmission of information and knowledge among all participants. Although families were not directly involved in the protocol’s design during this phase, their needs were addressed through the shared insights of NICU professionals, especially nurses, with extensive experience supporting parents in our unit. These observations, emerging through clinical practice and discussed in the focus group, informed the protocol’s structure. Future studies will directly involve families to formally assess their needs and expectations and contribute to the outcome selection. Moreover, although feedback from professionals regarding the project was positive, we did not measure the impact on the newborns’ ability to achieve independent oral feeding earlier or on the duration of hospitalization. Finally, as this was a quality improvement initiative and not a research study, no formal outcome measurements or statistical analyses were conducted.

## Conclusion

Preterm birth can have a profound impact on infant neurodevelopment. Early experiences are critical for brain maturation and shaping developmental trajectories. The implementation of early intervention programs is vital to support parent-infant bonding, foster positive sensory experiences, and mitigate environmental impacts on maturation and essential skills, such as effective autonomous feeding. The STAmmi VICINO protocol offers a highly adaptable pathway tailored to each child’s clinical condition and family needs. Led by the care team, particularly nurses, TNPEE, and speech therapists, it can promote parental empowerment and infant development. Its flexibility allows customization to support oral feeding and neurobehavioral skills, which are key elements for safe discharge. The protocol aims to support caregivers in their educational role during this delicate period, encouraging positive dyadic relationships. It also has the potential to facilitate communication between caregivers, parents, and within the team, promoting a multidisciplinary approach that supports neurodevelopmentally supportive care strategies for preterm newborns. While further evaluation is needed, this protocol may serve as a useful framework for structuring neurodevelopmentally supportive and family-centered care within NICU practice. Future efforts could focus on assessing its replicability and long-term outcomes across different settings.

## Data Availability

The original contributions presented in the study are included in the article/supplementary material, further inquiries can be directed to the corresponding author.

## References

[1] Osterman M Valenzuela C Hamilton B Driscoll A Martin J. Births: Final data for 2022. National Center Health Statistics. (2024) 73:1–56. doi: 10.15620/cdc:145588, PMID: 38625869

[2] Ohuma EO Moller A-B Bradley E Chakwera S Hussain-Alkhateeb L Lewin A. National, regional, and global estimates of preterm birth in 2020, with trends from 2010: a systematic analysis. Lancet. (2023) 402:1261–71. doi: 10.1016/s0140-6736(23)00878-4, PMID: 37805217

[3] Kiechl-Kohlendorfer U Peglow UP Reiter G Ralser E Trawöger R. Adverse neurodevelopmental outcome in preterm infants: risk factor profiles for different gestational ages. Acta Paediatr. (2009) 98:792–6. doi: 10.1111/j.1651-2227.2009.01219.x, PMID: 19191762

[4] Marret S Foix-L’Hélias L Matis J Fresson J Alberge C Marpeau L. Neonatal and 5-year outcomes after birth at 30–34 weeks of gestation. Obstet Gynecol. (2007) 110:72–80. doi: 10.1097/01.aog.0000267498.95402.bd, PMID: 17601899

[5] Szpecht D Szymankiewicz M Nowak I Gadzinowski J. Intraventricular hemorrhage in neonates born before 32weeks of gestation-retrospective analysis of risk factors. Childs Nerv Syst. (2016) 32:1399–404. doi: 10.1007/s00381-016-3127-x, PMID: 27236782 PMC4967094

[6] Volpe JJ. The encephalopathy of prematurity—brain injury and impaired brain development inextricably intertwined. Semin Pediatr Neurol. (2009) 16:167–78. doi: 10.1016/j.spen.2009.09.005, PMID: 19945651 PMC2799246

[7] Chung EH Brown KA Chou J. Neurodevelopmental outcomes of preterm infants: a recent literature review. Transl Pediatr. (2020) 9:S3–8. doi: 10.21037/tp.2019.09.10, PMID: 32206579 PMC7082240

[8] Sansavini A Guarini A Justice LM Savini S Broccoli S Alessandroni R. Does preterm birth increase a child’s risk for language impairment? Early Hum Dev. (2010) 86:765–72. doi: 10.1016/j.earlhumdev.2010.08.014, PMID: 20846796

[9] Zambrana IM Ystrom E Sengpiel V Vollrath ME Jacobsson B. Preterm birth and risk for language delays before school entry: a sibling-control study. Dev Psychopathol. (2020) 33:47–52. doi: 10.1017/s0954579419001536, PMID: 31896377 PMC7900651

[10] Bos AF Roze E Van Braeckel KNJA Hitzert MM Tanis JC. Development of fine motor skills in preterm infants. Dev Med Child Neurol. (2013) 55:1–4. doi: 10.1111/dmcn.12297, PMID: 24237270

[11] Zhao T Li H Zhang Y Lester B Hussain N Griffith T. Early-life factors associated with neurobehavioral outcomes in preterm infants during NICU hospitalization. Pediatr Res. (2022) 92:1695–704. doi: 10.1038/s41390-022-02021-y, PMID: 35338349 PMC9509490

[12] Inder TE Volpe JJ Anderson PJ. Defining the neurologic consequences of preterm birth. N Engl J Med. (2023) 389:441–53. doi: 10.1056/nejmra2303347, PMID: 37530825

[13] Fazzi E Micheletti S Galli J. Visual impairment: a common sequela of preterm birth. NeoReviews. (2012) 13:e542–50. doi: 10.1542/neo.13-9-e542

[14] Camerota M Lester BM. Neurobehavioral outcomes of preterm infants: toward a holistic approach. Pediatr Res. (2024) 97:1475–80. doi: 10.1038/s41390-024-03505-9, PMID: 39179875 PMC11846960

[15] Treyvaud K Anderson PJ Lee KJ Kidokoro H Ure A Doyle LW. Psychiatric outcomes at age seven for very preterm children: rates and predictors. J Child Psychol Psychiatry. (2013) 54:772–9. doi: 10.1111/jcpp.12040, PMID: 23347471 PMC3821531

[16] Anderson DE Patel AD. Infants born preterm, stress, and neurodevelopment in the neonatal intensive care unit: might music have an impact? Develop Med Child Neurol. (2018) 60:256–66. doi: 10.1111/dmcn.13663, PMID: 29363098

[17] Browne JV Ross ES. Eating as a neurodevelopmental process for high-risk newborns. Clin Perinatol. (2011) 38:731–43. doi: 10.1016/j.clp.2011.08.004, PMID: 22107901

[18] Jadcherla SR Khot T Moore R Malkar M Gulati IK Slaughter JL. Feeding methods at discharge predict long-term feeding and neurodevelopmental outcomes in preterm infants referred for gastrostomy evaluation. J Pediatr (2016) 181:125–130.e1. doi: 10.1016/j.jpeds.2016.10.065 27939123 PMC5724518

[19] Bazyk S. Factors associated with the transition to oral feeding in infants fed by nasogastric tubes. Am J Occup Ther. (1990) 44:1070–8. doi: 10.5014/ajot.44.12.1070, PMID: 2126165

[20] Pridham K Brown R Sondel S Green C Wedel NY Lai H-C. Transition time to full nipple feeding for premature infants with a history of lung disease. J Obstet Gynecol Neonatal Nurs. (1998) 27:533–45. doi: 10.1111/j.1552-6909.1998.tb02620.x, PMID: 9773365

[21] Morris BH Landry SH Miller-Loncar CL Denson SE Swank PR Smith KE. Feeding, medical factors, and developmental outcome in premature infants. Clin Pediatr (Phila). (1999) 38:451–7. doi: 10.1177/000992289903800802, PMID: 10456239

[22] Ibrahim C Chavez P Smith D Craig J Pineda R. Oral motor interventions used to support the development of oral feeding skills in preterm infants: an integrative review. Early Hum Dev. (2024) 198:106125. doi: 10.1016/j.earlhumdev.2024.106125, PMID: 39362153

[23] Burklow KA Valerius KS Mcgrath AM Rudolph C. Relationship between feeding difficulties, medical complexity, and gestational age. Nutr Clin Pract. (2002) 17:373–8. doi: 10.1177/0115426502017006373, PMID: 16215014

[24] Als H. Developmental care in the newborn intensive care unit. Curr Opin Pediatr. (1998) 10:138–42. doi: 10.1097/00008480-199804000-00004, PMID: 9608890

[25] Field TM. Stimulation of preterm infants. Pediatr Rev. (2003) 24:4–11. doi: 10.1542/pir.24-1-4, PMID: 12509539

[26] Peng N-H Bachman J Chang Y-S Jenkins R Chen C-H Wang T-M. Relationships between environmental stressors and stress biobehavioral responses of preterm infants in NICU. Adv Neonatal Care. (2013) 13:S2–S10. doi: 10.1097/anc.0000000000000023 24042180

[27] Lester BM Miller RJ Hawes K Salisbury A Bigsby R Sullivan MC. Infant Neurobehavioral Development. Semin Perinatol. (2011) 35:8–19. doi: 10.1053/j.semperi.2010.10.003, PMID: 21255702 PMC3168949

[28] Soleimani F Azari N Shahrokhi A Ghiasvand H Fatollahierad S Rahmani N. Do NICU developmental care improve cognitive and motor outcomes for preterm infants? A systematic review and meta-analysis. BMC Pediatr. (2020) 20:67. doi: 10.1186/s12887-020-1953-1, PMID: 32054469 PMC7017495

[29] Als H. Toward a synactive theory of development: promise for the assessment and support of infant individuality. Infant Ment Health J. (1982) 3:229–43. doi: 10.1002/1097-0355(198224)3:4<229::aid-imhj2280030405>3.0.co;2-h

[30] Westrup B. Newborn individualized developmental care and assessment program (NIDCAP) — family-centered developmentally supportive care. Early Hum Dev. (2007) 83:443–9. doi: 10.1016/j.earlhumdev.2007.03.006, PMID: 17459617

[31] European Standards of care for newborn health. (2024) Available online at: https://newborn-health-standards.org/ [Accessed December 20, 2024].

[32] Whetten CH. Cue-based feeding in the NICU. Nurs Womens Health. (2016) 20:507–10. doi: 10.1016/j.nwh.2016.08.006, PMID: 27719780

[33] Monterosso L Holland BG Sly PD Mulcahy M Kristjanson L White K. The role of the neonatal intensive care nurse in decision-making: advocacy, involvement in ethical decisions and communication. Int J Nurs Pract. (2005) 11:108–17. doi: 10.1111/j.1440-172x.2005.00512.x, PMID: 15853789

[34] Brødsgaard A Pedersen JT Larsen P Weis J. Parents’ and nurses’ experiences of partnership in neonatal intensive care units: a qualitative review and meta-synthesis. J Clin Nurs. (2019) 28:3117–39. doi: 10.1111/jocn.14920, PMID: 31112337

[35] Barbosa VM. Teamwork in the neonatal intensive care unit. Phys Occup Ther Pediatr. (2013) 33:5–26. doi: 10.3109/01942638.2012.729556, PMID: 23311520

[36] Cerroni F Pirozzi A Guarino A Albano PA Martino F Bonifacio A. Developmental specialists in Europe: comparison between healthcare and education systems, and rehabilitation in child disability. J Adv Health Care. (2024) 6. doi: 10.36017/jahc202463379

[37] Purpura G Coratti G. Neuro and psychomotor therapist of developmental age professional in Italy: an anomaly or an opportunity? Arch Rehab Res Clinic Translation. (2024) 6:100372. doi: 10.1016/j.arrct.2024.100372, PMID: 39822210 PMC11734002

[38] Lessen BS. Effect of the premature infant Oral motor intervention on feeding progression and length of stay in preterm infants. Adv Neonatal Care. (2011) 11:129–39. doi: 10.1097/anc.0b013e3182115a2a, PMID: 21730902

[39] Beissel A Tume LN Hommey S Pillet F Gauthier-Moulinier H Denis A. Impact of a nurse education programme on oral feeding in a neonatal unit. Nurs Crit Care. (2022) 29:287–95. doi: 10.1111/nicc.12840, PMID: 36054567

[40] American Academy of Pediatrics Committee on Fetus and Newborn. Hospital discharge of the high-risk neonate. Pediatrics. (2008) 122:1119–26. doi: 10.1542/peds.2008-2174 18977994

[41] Griffin IJ Lee HC Bertino E Profit J Tancredi DJ. Postnatal growth failure in very low birthweight infants born between 2005 and 2012. Arch Dis Child Fetal Neonatal Ed. (2015) 101:50–5. doi: 10.1136/archdischild-2014-308095, PMID: 26201534

[42] Lau C. Development of infant oral feeding skills: what do we know? Am J Clin Nutr. (2016) 103:616S–21S. doi: 10.3945/ajcn.115.109603, PMID: 26791183 PMC4733254

[43] Lau C. To individualize the Management Care of High-Risk Infants with Oral Feeding Challenges: what do we know? What can we do? Front Pediatr. (2020) 8:296. doi: 10.3389/fped.2020.00296, PMID: 32582596 PMC7297031

[44] Kinneer MD Beachy P. Nipple feeding premature infants in the neonatal intensive-care unit: factors and decisions. J Obstetric Gynecologic Neonatal Nursing. (1994) 23:105–12. doi: 10.1111/j.1552-6909.1994.tb01859.x, PMID: 8201452

[45] Siddell EP Froman RD. A national survey of neonatal intensive-care units: criteria used to determine readiness for oral feedings. J Obstetric Gynecologic Neonatal Nursing. (1994) 23:783–9. doi: 10.1111/j.1552-6909.1994.tb01953.x, PMID: 7853084

[46] Lyu T Zhang Y Hu X Lau C Li L Gu Y. Management of oral feeding challenges in neonatal intensive care units (NICUs): a national survey in China. Front Pediatr. (2020) 8:336. doi: 10.3389/fped.2020.00336, PMID: 32671001 PMC7328344

[47] Crowe L Chang A Wallace K. Instruments for assessing readiness to commence suck feeds in preterm infants: effects on time to establish full oral feeding and duration of hospitalisation. Cochrane Database Syst Rev. (2017) 2017:CD005586. doi: 10.1002/14651858.cd005586.pub3, PMID: 22513933

[48] Grabill M Ibrahim C Smith J Pineda R. Prevalence of early feeding alterations among preterm infants and their relationship to early neurobehavior. Am J Occup Ther. (2023) 77:7703205170. doi: 10.5014/ajot.2023.050123, PMID: 37253183

[49] Thurin S Ruediger J Mohsini K Cho R. Impact of nurse-regulated feedings on growth velocity and weight gain of 1200-1500 g preterm infants. J Clin Neonatol. (2012) 1:21–4. doi: 10.4103/2249-4847.92243, PMID: 24027680 PMC3761988

[50] Aykanat Girgin B Gözen D. Turkish neonatal nurses’ knowledge and practices regarding the transition to oral feeding in preterm infants: a descriptive, cross-sectional study. J Pediatr Nurs. (2020) 53:e179–85. doi: 10.1016/j.pedn.2020.03.017, PMID: 32321668

[51] Pineda R Misikoff M Ghahramani S Smith J Mathur A. Description and evidence on the supporting and enhancing neonatal intensive care unit sensory experiences (SENSE) program. Acta Paediatr. (2025) 114:731–42. doi: 10.1111/apa.17293 38809111 PMC11894784

[52] White-Traut R Rankin KM Pham T Li Z Liu L. Preterm infants’ orally directed behaviors and behavioral state responses to the integrated H-HOPE intervention. Infant Behav Dev. (2014) 37:583–96. doi: 10.1016/j.infbeh.2014.08.001, PMID: 25189523 PMC4262744

